# In Utero Fetal Ovarian Torsion with Imaging Findings on Ultrasound and MRI

**DOI:** 10.1155/2012/151020

**Published:** 2012-07-12

**Authors:** Ripple Sheth, Dennis Hoelzer, Emily Scattergood, Pauline Germaine

**Affiliations:** ^1^Department of Diagnostic Radiology, Cooper University Hospital, 1 Cooper Plaza, Suite B23, Camden, NJ 08103, USA; ^2^Department of Pediatric Surgery, Cooper University Hospital, 1 Cooper Plaza, Suite B23, Camden, NJ 08103, USA

## Abstract

Early diagnosis of ovarian torsion is critical in avoiding complications and planning management. Therefore, it is important to understand and assess the imaging findings of ovarian torsion. Ultrasound is the imaging modality of choice; however, it is not always definitive and diagnosis can be challenging. MRI is a better imaging modality to evaluate for signs of complications and to arrive at a more definitive diagnosis. We present a case of in utero ovarian torsion diagnosed during routine prenatal ultrasound with imaging findings on ultrasound and MRI postnatally.

## 1. Introduction

The incidence of fetal ovarian cysts (FOCs) detected in utero has increased in the past decade due to the availability and prevalent use of ultrasound [1]. FOCs are usually seen towards the end of the second trimester during a routine prenatal ultrasound. Stimulation of the fetal ovary by placental and maternal hormones leads to the development of ovarian cysts. They tend to regress shortly after birth once the hormonal stimulation has decreased [2, 3]. Complications such as hemorrhage, rupture, and torsion can develop. Ovarian torsion is the most common complication of an untreated ovarian cyst [1, 2].

According to the Nussbaum's criteria, cysts can be classified as “simple” or “complex” based on their ultrasonographic features. Simple cysts can be referred to as follicular cysts, which are completely anechoic on ultrasound. Complex cysts can have an echogenic wall, internal septae, fluid-debris level, or a blood clot. Complicated cysts tend to be more concerning for an underlying ovarian torsion. Simple cysts are typically the result of benign stimulation and can regress if less than 4.0 cm. Larger cysts, classified as greater than 4.0 cm, have susceptibility for torsion and surgical treatment is recommended [4].

Diagnosis of ovarian torsion is critical for timely management and is easily detectable with the use of ultrasound. This paperis unique because of the classic and identical imaging findings of ovarian torsion seen both on ultrasound and MRI. There have been no reported cases in the literature regarding MRI findings on in utero ovarian torsion.

## 2. Case Report

A 27-year-old G_11_ P_2262_ female with no significant past medical history had a routine prenatal ultrasound at 35 weeks gestation. The ultrasound showed a 4.0 cm cystic lesion in the right ovary of the fetus. The remainder of the pregnancy was uneventful. The female neonate was delivered vaginally without any complications.

**Figure 1:**
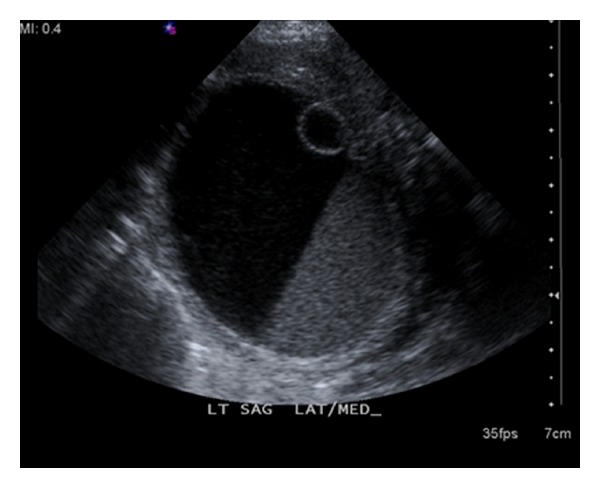
A sagittal gray scale image of the left ovary shows a 4.5 × 5.6 × 4.5 cm cystic mass with low-level-dependent echoes, consistent with internal hemorrhage. A smaller cyst is seen internally, measuring 1.0 cm in diameter. The findings were identical when compared to the prenatal ultrasound. Similar findings were seen on the parental ultrasound.

**Figure 2:**
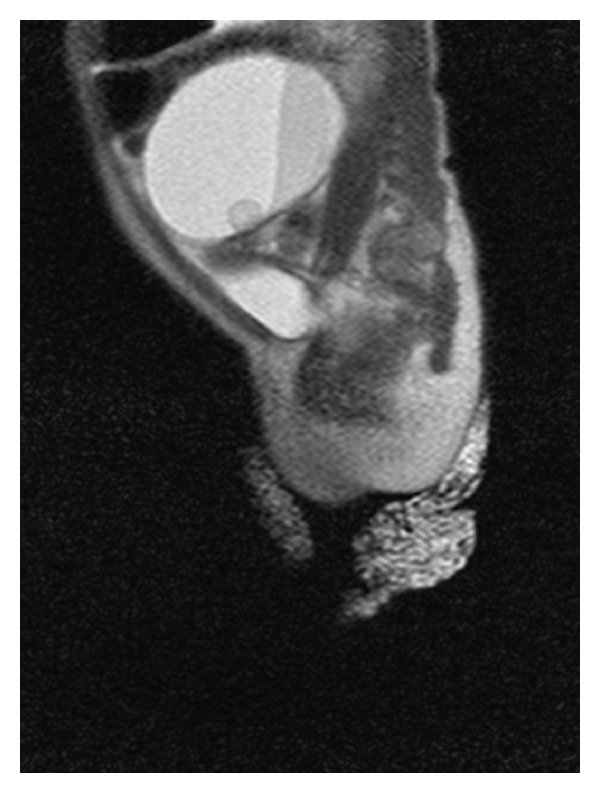
A haste sagittal MBH MRI image demonstrates a 4.5 × 5.6 × 4.5 cm cystic mass with layering hemorrhagic or proteinaceous elements within it. A nodular internal 1.0 cm cystic portion against the inner wall of the large mass is also seen.

A pelvic ultrasound was performed one day after birth to follow up on the ovarian cyst seen in utero. Ultrasound showed a 5.8 × 4.0 × 4.3 cm cystic mass with internal hemorrhage. There was a smaller cyst internally measuring 0.8 × 0.9 × 1.0 cm (Figure 1). An MRI was recommended for further characterization, which was performed on the same day. MRI showed a 4.5 × 5.6 × 4.5 cm cystic mass with layering hemorrhage or proteinaceous elements. A 1.0 cm nodular cyst was present against the inner wall of the larger cyst (Figure 2). The size of the ovarian cyst had increased in size with morphologic changes since the prenatal ultrasound. Suspicion for ovarian torsion was high and the female newborn underwent oopherectomy on the seventh day of life. Imaging findings of ovarian torsion with internal hemorrhage were confirmed in the surgical pathology report.

## 3. Discussion

Fetal ovarian cysts are the most common abdominal masses in fetuses and neonates. Majority of them are benign and resolve within a few months after birth. The earliest age at which a FOC can be detected is at 19th week of gestation. However, most cysts are identified around 28 weeks of gestation [2].

The etiology of simple follicular cysts is thought to occur from stimulation by maternal and placental chorionic gonadotropin levels. The incidence increases in complicated pregnancies with mothers with Rh incompatibility, diabetes mellitus, and preeclampsia. In preterm infants, the gonadostat mechanism is immature and can lead to ovarian hyperstimulation. Fetal hypothyroidism and congenital adrenal hyperplasia have also been reported as causes of fetal ovarian cysts [4, 5]. Once the maternal-placental estrogens and beta-human chorionic gonadotropin (B-hCG) decrease after birth, the cysts can spontaneously regress. However, since follicle-stimulating hormone (FSH) and luteinizing hormone (LH) levels rise until the gonadostat mechanism matures, cysts can continue to enlarge or persist until about three months after birth [5]. During this period, complications such as hemorrhage, rupture, or torsion can occur [1, 2].

Ovarian torsion results from either partial or complete twist of the ovary and fallopian tube. Initially, there is compromise of lymphatic drainage leading to lymphatic edema, which causes enlargement of the ovary. Venous obstruction and hemorrhagic infarction can follow. Eventually, arterial blood supply will be compromised, which can result in gangrene, infection, and peritonitis. Torsion can occur in normal ovaries; however, an underlying ovarian mass can increase the risk of torsion. Neonatal ovarian cysts can clinically cause pain, vomiting, fever, irritability, and abdominal distention. Untreated ovarian torsion can lead to the loss of ovary and infertility [1, 4]. Therefore, timely diagnosis is critical to prevent complications. Ovarian torsion is rarely seen in the postnatal period and more frequently occurs in utero and during birth. Hence, sonographic follow up throughout gestation is highly recommended.

Ultrasound is the imaging modality of choice to evaluate for ovarian torsion due to easy availability and ease of use. When torsion of a cyst occurs, its size increases rapidly and ultrasound features change to complex. Complex cysts will have debris, internal septae, or hemorrhage. Multiple cortical follicles at the periphery in a unilaterally enlarged ovary have also been reported [1]. Multifollicular enlargement occurs from transudation of fluid into the follicles secondary to congestion from circulatory compromise in torsion. Color and spectral Doppler ultrasound can be used to evaluate ovarian vascularity. Research has shown that absence of blood flow is indicative of torsion, however; presence of blood flow does not exclude torsion. As circulatory impairment occurs in torsion, transudation of fluid and hemorrhage into follicles occurs [1]. Nussbaum et al. [4] concluded that ultrasound findings of fluid-debris level with peripheral cyst occurred only in complicated, twisted cysts. When clinical suspicion for torsion is high and ultrasound findings are equivocal, MRI can be performed to better evaluate for torsion. Findings of complicated cysts described above are easier to assess on MRI.

In conclusion, serial ultrasound examinations of fetal ovarian cysts are critical since ovarian torsion is most common in the intrauterine period [3]. When small and simple follicular cysts start to enlarge rapidly with changes in ultrasound features, ovarian torsion should be highly suspected. Unilaterally enlarged ovary with peripheral cysts containing fluid-debris level is highly suspicious for ovarian torsion and hemorrhagic infarction. MRI is a useful imaging modality and should be performed when ultrasound does not provide definitive information or when serial ultrasound examinations do not show spontaneous regression of ovarian cysts.
